# Morphology and Chemical Composition of the Nuchal Glands of Indonesian Snakes With a Description of a Novel Type of Glands

**DOI:** 10.1002/jmor.70071

**Published:** 2025-08-14

**Authors:** Syahfitri Anita, Takato Inoue, Aya Inoue, Koshiro Eto, Amir Hamidy, Naoki Mori, Akira Mori

**Affiliations:** ^1^ Department of Zoology, Graduate School of Science Kyoto University, Sakyo Kyoto Japan; ^2^ Museum Zoologicum Bogoriense, Research Center for Biosystematic and Evolution National Research and Innovation Agency (BRIN) Bogor Indonesia; ^3^ Department of Applied Life Science, Graduate School of Agriculture Kyoto University, Sakyo Kyoto Japan; ^4^ Current address: Department of Applied Biosciences, Graduate School of Bioagricultural Sciences Nagoya University Chikusa Nagoya Japan; ^5^ Kitakyushu Museum of Natural History and Human History Fukuoka Japan

**Keywords:** bufonidae, bufotoxin, chemical defence, natricinae, *Rhabdophis*

## Abstract

Several species of natricine snakes sequester bufadienolides from toads, store them in their nuchal glands, and reutilize them for their defense. This study aimed to examine the presence and morphological features of nuchal glands of natricine snakes distributed in Indonesia, containing several lineages of this group. When the presence of the glands was confirmed, the gland fluids were analyzed to identify their chemical components. Secretions from the parotoid glands of several species of toads in Indonesia were also analyzed. Morphological examination of the glands was conducted by observing the presence or absence of structures and recording the spatial pattern of the glands. The glandular fluids of three snake species and four toad species were extracted and analyzed by liquid chromatography/mass spectrometry (LC/MS). Nuchal glands or organs similar to the glands were found on the head or neck region of preserved or freshly dead specimens of *Rhabdophis subminiatus*, *R. flaviceps*, *R. rhodomelas,* and *Rhabdophis* spp. of Sulawesi, although such a gland was not found in the other genera of natricine snakes. These *Rhabdophis* species have different spatial patterns of glands, and particularly *Rhabdophis* spp. of Sulawesi showed an unusual novel gland form in the temporal and occipital regions of the head, possibly comparable to the nuchal glands. Bufadienolides of the bufogenin and bufotoxin types were identified from all toad gland fluids. In contrast, the glandular fluids of snakes, *R. subminiatus*, *R. flaviceps*, and *Rhabdophis* spp. of Sulawesi store only bufogenin‐type bufadienolides. Bufadienolide profiles of snakes and toads indicate that the toxin composition is highly diverse among species. The results suggest that snakes would be able to chemically convert dietary bufadienolides from toads and reutilize some bufadienolides readily. This study demonstrated that the form and location of “nuchal glands” in a snake's body are more diverse than previously recognized.

## Graphical abstract

Nuchal glands, or organs resembling glands, were found in the head or neck region of *Rhabdophis* spp. from Indonesia. These species exhibit different spatial patterns of glands, with *Rhabdophis* from Sulawesi in particular showing a novel form located in the temporal and occipital regions of the head—possibly comparable to nuchal glands. This study demonstrates that the form and location of “nuchal glands” in snake bodies are more diverse than previously recognized.

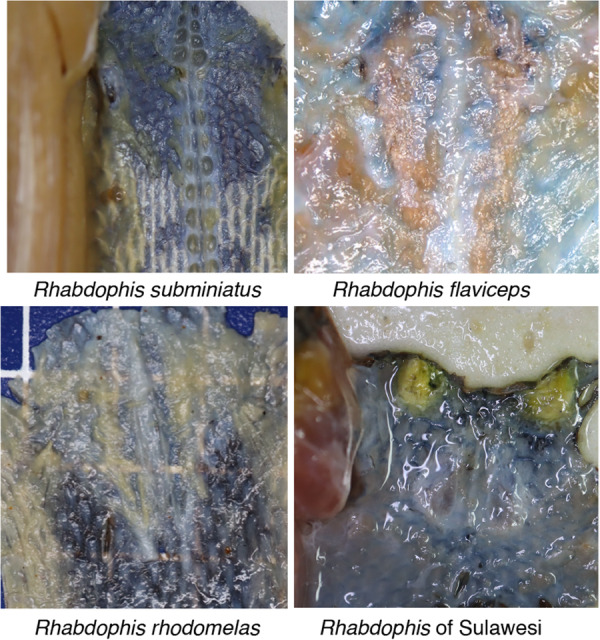

## 1 Introduction

Chemical defense is widespread among animals, and the compounds involved may be either synthesized by the animals themselves or acquired from environmental sources. Bufonid toads synthesize toxins of bufadienolides (BDs) in their paired parotoid glands for defense against natural enemies (Hutchinson and Savitzky 2004). Bufadienolides are cardioactive C‐24 steroids and are distinguished from other cardiotonic steroids by the presence of a pyrone ring, and a six‐membered lactone ring, at the C‐17 position on the steroid nucleus. Bufadienolides of toads are usually classified into bufogenin, characterized by the presence of a hydroxyl in C‐3, and bufotoxin, which is differentiated by a suberoyl‐l‐arginyl at the same position (Zou et al. 2022). Bufadienolides are toxic molecules, but many species of snakes and large predators have evolved the ability to overcome the lethal effects of BDs and thus are able to readily feed on toxic toads (Ujvari et al. 2015). Furthermore, several snake species can prey on toxic toads, sequester the BDs, and reutilize them in their own defense. This is seen in snakes with nuchal glands belonging to the natricine genus *Rhabdophis* (Mori et al. 2012).

The nuchal glands are organs arranged in pairs on each side of the mid‐dorsal scales of the nuchal region and are embedded in the dermis without openings or ducts, and rupture of the skin discharges fluids from the glands (Nakamura 1935). The glands are not always confined to the neck region but, in some species, are extended throughout the whole length of the body (nucho‐dorsal glands; Smith 1938). More detailed studies confirmed the presence of BDs in nuchal gland fluids of *R. tigrinus* and demonstrated the inability of the snake to biosynthesize the toxins themselves but the ability to sequester them from toads (Akizawa et al. 1985; Hutchinson et al. 2007, 2012; Mori et al. 2012). Currently, approximately 20 species of snakes are known to possess nuchal or nucho‐dorsal glands (Mori et al. 2023). Detailed morphological examinations on species with the nucho‐dorsal glands of *R. adleri*, *R. nuchalis*, *R. pentasupralabialis*, *R. guangdongensis*, and *R. plumbicolor* revealed differences in spatial arrangement, position, and size of the glands (Mori et al. 2016a, 2016c; Zhu et al. 2020).

Among Indonesian *Rhabdophis*, the nuchal glands are known to be present in *R. subminiatus*, *R. flaviceps*, and *R. rhodomelas* and absent in *R. chrysargos* and *R. conspicillatus* (Smith 1938; Mori et al. 2012, 2016b). Since Smith's (1938) observations, there has been no detailed study of the structure of nuchal glands in Indonesian snakes, and the chemical composition of the fluids in their glands is completely unknown. In addition, there are several other natricine species known to be distributed in Indonesia, such as *R. chrysargoides* and *R. callistus*, which are native to Sulawesi, and the nature of their nuchal glands is completely unknown. Furthermore, the toxin composition of skin secretions from toads distributed in Indonesia is also unknown. Studies summarizing the toxins of the skin secretions of toads are available in Krenn and Kopp (1998), Li et al. (2021), and Zou et al. (2022), but the locality of these toads was not specified. Bufadienolides profiles of toads and snakes may provide hints regarding the potential metabolism of BDs synthesis and may further enhance the understanding of the toxin sequestration system of the snakes. Here, we studied the nuchal glands of natricine snakes distributed in Indonesia, specifically by examining their gland structure to reveal the morphological features and spatial arrangements. We also performed a chemical analysis of the secretions from the parotoid glands of toads in Indonesia and the fluids from the nuchal glands or similar organs of Indonesian snakes to determine the presence and composition of BDs.

## 2 Materials and Methods

### 2.1 Samples and Specimen Identification

All specimens in this study belong to the natricine subfamily of Colubridae, which includes snakes in the genera *Rhabdophis*, *Xenochrophis*, *Fowlea*, and *Tropidonophis*. The taxonomic status of *Rhabdophis* from Sulawesi, *R. chrysargoides* and *R. callistus*, is problematic because they have overlapping morphological characters and distribution, although differences in juvenile coloration have been suggested (Lang and Vogel 2005). In the present study, we tentatively combined the morphological and glandular information for *R. chrysargoides* and *R. callistus* specimens originating from various locations in Sulawesi Island, and refer to them as *Rhabdophis* spp. of Sulawesi or Sulawesian *Rhabdophis*. The sample sizes and localities for specimens are listed in Table 1. Specimens examined were in freshly dead or preserved condition. Most of the preserved specimens were obtained from the Museum Zoologicum Bogoriense (MZB) collection and were used for examination of gland morphology. Initial species identification was based on the locality and common body pattern, as described in the literature (Das 2018; Lang and Vogel 2005; Lang 2017). Further species identification was based on the diagnostic morphological characters (Supporting Information S1: Table S1). The dorsal scale rows (DSR) were counted at mid‐body, and the number of ventral scales (VEN) was counted according to Dowling (1951). Ventral scales were also used as landmarks for determining the positions of glands on the body. Subcaudal scales were counted as the number of scales from the cloaca to the tail tip, and the unpaired terminal scale of the tail was not included. Scales on the head were counted on both sides of the head, but presented only for the left side. Snout‐vent length (SVL) and tail length (TL) were measured to the nearest 1 mm. We also collected toads from the snake collection site to extract their gland fluid. *Phrynoidis asper* was collected from the western and central regions of Java Island. *Duttaphrynus melanostictus* was collected from the western and central regions of Java Island, as well as the central area of Sulawesi Island. One specimen of *Ingerophrynus biporcatus* was collected from the Yogyakarta region of Java. Specimens of *Ingerophrynus celebensis* were collected from the central region of Sulawesi Island.

**Table 1:** Species, sample size, and locality of snakes used to examine gland features. The sample size is shown in parentheses.

Species	Condition		Locality	
	Freshly dead	Preserved specimens	Island	City/Region
*Rhabdophis subminiatus*	Male (1)	Female (4), male (3)	Java	Magelang, Cilacap, Karawang, Banyuwangi, Ujungkulon
*Rhabdophis* of Sulawesi	Female (1)	Male (5), female (1)	Sulawesi	Poso, Parigi Moutong, Kolaka, Buton, Nantu
*Rhabdophis flaviceps*	Female (5), male (3)	Female (2), male (1)	Kalimantan	Kumai, Landak, Kutai
			Sumatra	Jambi, Lampung
*Rhabdophis rhodomelas*	—	Female (2), male (1)	Sumatra	Lampung
*Rhabdophis chrysargos*	—	Female (2)	Sumatra	unknwon
			Java	Cibodas
*Xenochrophis trianguligerus*	—	Female (2)	Sulawesi	Maros
			Java	Bogor
*Fowlea melanzostus*	—	Female (1), male (2)	Java	Bogor, Pati, Tuban
*Tropidonophis mairii*	—	Female (1)	Papua	Jaya Wijaya
*Tropidonophis doriae*	—	Male (1)	Papua	Siewa

### 2.2 Morphological Examination of the Glands

Morphological examination was conducted to investigate the features of the nuchal glands or similar organs. We followed the method described in previous studies, with some modifications (Mori et al. 2016a, 2016c). The dorsal skin was carefully peeled off from the anterior edge (or posterior edge in several species) of the parietal scales to the neck region using scissors and a scalpel to find any structure resembling nuchal glands, referring to descriptions, drawings, or photographs in published articles (Smith 1938; Mori et al. 2012, 2016a, 2016c). The presence or absence of structures, the type of glands (sacculated or unsacculated), the size of glands, and the number of gland pairs were recorded. The type of gland was defined according to Smith (1938), in which sacculated glands are a series of spherical or oval structures arranged in regular chains on either side of the vertebral line, whereas an unsacculated gland is a continuous piece of tissue that lies beneath the areas of elastic skin, which can be discovered by laterally stretching the neck skin and was called as “naked skin” by Smith (1938). For the sacculated type, maximum length (measured parallel to the longitudinal body axis) and maximum width (measured perpendicular to the major body axis) of a gland were measured to the nearest 0.05 mm using calipers. In addition, for *R. subminiatus*, the size of the first (anteriormost), middle (the 6th, 7th, or 8th row), and last (posteriormost) glands was measured. For the unsacculated glands, the maximum length and width of the unusual structural area, which seemed to be a peculiar organ similar to the nuchal glands, were measured. The position of the glands in relation to body scale rows was recorded as follows: the mid‐dorsal scale row, along the vertebral line, was defined as zero, and the number of each scale row was counted in the ventrolateral direction until reaching the scale under which the glands were positioned.

### 2.3 Extraction of Glandular Fluid

The fluid of the nuchal glands or similar organs was extracted from live or freshly dead adult individuals of *R. subminiatus*, *R. flaviceps*, and *Rhabdophis* spp. Sulawesi (see Table 2 for details). Several individuals of *R. subminiatus* were kept for less than a year, fed on *Fejervarya* spp., and used in behavioral experiments before their glandular fluid was extracted. The fluid was obtained by gently squeezing the dorsal surface of the neck region using a Kimwipe (Kimtech Science) while wearing new nitrile gloves for each individual. Parotoid gland secretions from adult toads representing the species *Duttaphrynus melanostictus*, *Phrynoidis asper, Ingerophrynus biporcatus*, and *I. celebensis* (see Table 2) were collected by manually squeezing the glands using a Kimwipe (Kimtech Science). At the end of each sampling time, a Kimwipe without fluid was prepared as a control. Each Kimwipe was inserted into a glass vial filled with approximately 3 mL of methanol (MeOH), sealed with a Teflon‐lined cap, and stored at −20°C in the dark for further analysis. The immersed Kimwipe was washed with 5 mL of MeOH, and then both the storage and wash solutions of MeOH were combined and filtered with a syringe filter (DISMIC‐13HP, pore diameter, 0.45 μm; Toyo Roshi Kaisha Ltd., Tokyo, Japan). The filtrate was concentrated to dryness under reduced pressure. The crude extract (ext.) was weighed, and dissolved in MeOH at a concentration of 1–10 mg ext./mL. This MeOH solution was combined with digitoxigenin (as an internal standard, IS) and diluted to a concentration of 100 ng ext./μL and 2.5 ng/μL of IS.

**Table 2:** Sample size and locality of snakes and toads used for chemical analyses of the gland fluids.

Species	Sample size (individuals)	Locality
*Rhabdophis subminiatus*	16	West Java, Central Java
*Rhabdophis flaviceps*	4	West Kalimantan, Central Kalimantan,
*Rhabdophis* of Sulawesi	6	Central Sulawesi
*Duttaphrynus melanostictus*	11	West Java, Central Java, Central Sulawesi
*Phrynoidis aspera*	7	West Java, Central Java
*Ingerophrynus biporcatus*	1	Central Java
*Ingerophrynus celebensis*	12	Central Sulawesi

### 2.4 LCMS‐IT‐TOF Analysis

LCMS‐IT‐TOF analysis was performed using a combination of a Prominence HPLC system (Shimadzu Co., Kyoto, Japan) and LCMS‐IT‐TOF (Shimadzu Co., Kyoto, Japan). The LC system consisted of a system controller (CBM‐20A), pump (LC‐20AD), autosampler (SIL‐ 20AC), column oven (CTO‐20AC), and online degasser (DGU‐20A3). LCMS solutions Ver. 3.60 (Shimadzu Co., Kyoto, Japan) was used for data collection and analysis. Compounds were separated on a reversed‐phase column (Mightysil RP‐18 GP 50 mm × 2.0 mm I.D., particle size 5 μm; Kanto Chemical Co). Elution was performed with a gradient of 20% (0–2 min), 20%–55% (2–20 min), 55%–100% (20–35 min), and 100% (35–40 min) MeOH in H_2_O containing 0.1% (v/v) formic acid. The column oven temperature was maintained at 40°C. MS was run in atmospheric chemical ionization (APCI) positive ion mode, nebulizer gas flow rate 2.5 L/min, probe voltage 4.5 kV, probe temperature 400°C, curved desolvation line (CDL) temperature 250°C, heat block temperature 200°C, detector voltage 1.9 kV, cooling gas pressure 97 kPa, and collision gas pressure 36 kPa. Argon was used as the cooling gas and collision gas. The range of *m/z* values scanned in MS was 350–1000, with 10 repeats and an ion accumulation time of 10.0 msec. The presence of compounds was determined by introducing 2 μL of the sample prepared to a concentration of 100 ng (ext.)/μL into LCMS and analyzing the extracted ion chromatogram (XIC) for the presence or absence of peaks and of the UV (ultraviolet) light of 300 nm wavelength due to the pyrone ring, a common structure in BDs. Each BD was tentatively named as a combination of a number and a lowercase letter; the number is the *m/z* value of the predicted proton adducted ion ([M + H]^+^), and the letter, in alphabetical order, was assigned to represent the elution order of BDs possessing the same *m/z* value. The chemical structure estimation was made by referring to previous studies, and the compound with the predicted chemical structure was named following the common name or the combination of numbers and uppercase letters in parallel to the previous studies (Steyn and van Heerden 1998; Inoue et al. 2020, 2021). To analyze the intersections between identified BDs among species, we used the UpSet plot, a visualization technique for the quantitative analysis of interactive sets through Rstudio (Lex et al. 2014; Conway et al. 2017; RStudio Team 2020).

## 3 Results

The specimens examined were identified as *Rhabdophis subminiatus*, *R. flaviceps*, *R. rhodomelas*, *Rhabdophis* spp. of Sulawesi (*R. chrysargoides* or *R. callistus*), *R. chrysargos*, *Xenochrophis trianguligerus*, *Fowlea melanzosta*, *Tropidonophis doriae*, and *T. mairii* (Table 3). There was no trace of glands or similar structures observed in the interior of the parietal‐neck skin of *R. chrysargos*, *X. trianguligerus*, *F. melanzostus*, *T. doriae*, and *T. mairii* (Supporting Information S1: Figure S1). Therefore, we concluded that these species do not have nuchal glands. Nuchal glands, unusual structures resembling glands, or traces of glands were found on the head or neck region of preserved or freshly dead specimens of *R. subminiatus*, *R. flaviceps*, *R. rhodomelas*, and *Rhabdophis* spp. of Sulawesi.

**Table 3:** Body size, scale counts, and the occurrence of glands in Indonesian natricine snakes. The sample size is shown in parentheses after the specimen condition. Mean ± SD are shown, and the range is displayed below the mean.

Species	Specimen condition	SVL (mm)	TL (mm)	DSR	VEN	SC	SL	Gland occurrence
*Rhabdophis subminiatus*	Freshly dead (1), Preserved (7)	462 ± 35.01	145 ± 39.83	19	144 ± 6.42	62 ± 12.90	8	Present
		426–522	109–237		136–158	41–74		
*Rhabdophis* of Sulawesi	Freshly dead (1), Preserved (6)	555 ± 150.39	151 ± 27.66	21	148 ± 1.79	62 ± 4.74	9	Present
		369–787	12.1–17.7		143–155	55–70		
*Rhabdophis flaviceps*	Freshly dead (8), Preserved (3)	536 ± 173.12	129 ± 36.22	19	123 ± 2.56	46 ± 6.44	8–9	Present/absent
		252–751	61–164		121–127	36–55		
*Rhabdophis rhodomelas*	Preserved (3)	405 ± 37.09	97 ± 11.14	19	136 ± 1.53	42 ± 10.81	7–8	Present/absent
		365–438	87–109		134–137	30–51		
*Rhabdophis chrysargos*	Preserved (2)	583 ± 152.03	203 ± 8.49	19	151 ± 1.41	78 ± 11.31	9	Absent
		476–691	197–209		144–150	70–86		
*Xenochrophis trianguligerus*	Preserved (2)	687 ± 79.19	288 ± 70	19	139 ± 3.53	81 ± 8.49	9	Absent
		631–743	239–338		136–141	75–87		
*Fowlea melanzostus*	Preserved (3)	660 ± 133.38	210 ± 23.13	19	135 ± 6.66	62 ± 12.74	9	Absent
		581–814	189–235		128–140	48–71		
*Tropidonophis. mairii*	Preserved (1)	580	209	15	160	78	8	Absent
*Tropidonophis doriae*	Preserved (1)	721	213	17	144	66	8	Absent

Abbreviations: DSR, number of scale rows at midbody; SC, number of subcaudal pairs; SD, standard deviation; SL, number of supralabials (left, range was displayed for species having variation); SVL, snout‐vent length (mm); TL, tail length (mm); VEN, number of ventral scales.

### 3.1 Rhabdophis subminiatus

**Figure 1:**
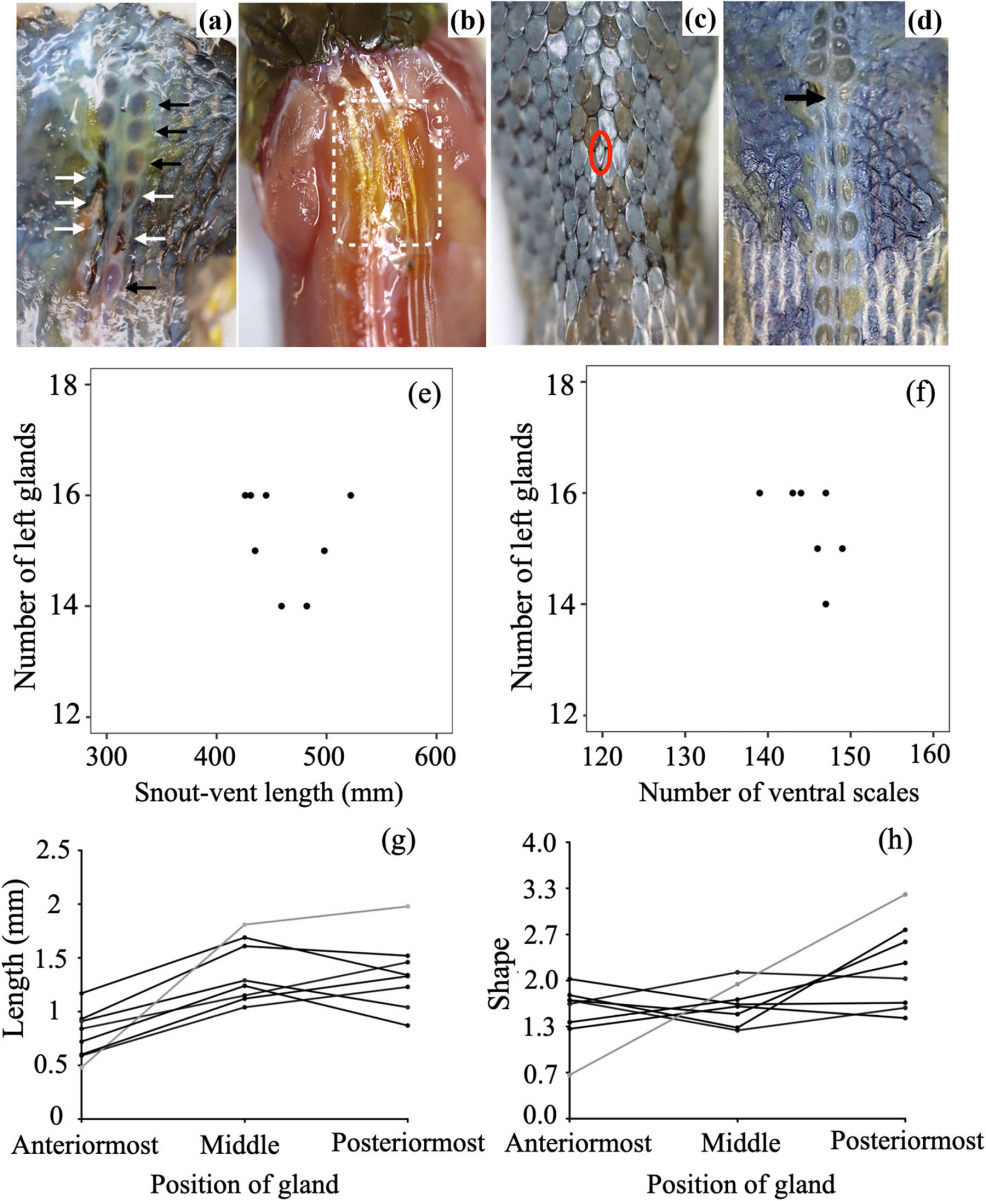
The sacculated nuchal glands of *Rhabdophis subminiatus*. (a) The nuchal glands of freshly dead specimens; white and black arrows indicate broken glands and glands with fluid, respectively. (b) Some parts of the neck muscle turned yellowish caused by the gland fluid (white box). (c) A scale appears in the vertebral line of *R. subminiatus* (red circle), starting a new mid‐body dorsal scale row. (d) This new scale row starting after the black arrow corresponds to the different shape and position of glands (compare between the anterior and posterior to the arrow). (e) Relationships between the number of left glands observed in eight specimens of *R. subminiatus* with its snout‐vent length. (f) Relationships of the number of left glands observed in eight specimens of *R. subminiatus* and its number of ventral scales. (g) Changes in the length of glands in the left row along the body axis. (h) Changes in the shape (length/width) of glands in the left row along the body axis. Grey lines (in g and h) indicate measurement of glands containing fluid in freshly dead specimen.

All specimens possessed the sacculated type of nuchal glands in the neck skin region. The gland fluid was transparent when it filled the gland (Figure 1a), but changed to yellowish after it oozed out from the gland (Figure 1b). The chains of glands resided on both sides of the vertebral line in symmetrical pairs, starting near the posterior edge of the parietal plates, which corresponded to the first or second ventral scale, and extended to the 12th–16th ventral scales. The position of the gland pair in each row corresponded to specific dorsal scale rows and can be divided into two parts based on their position relative to a new row of mid‐dorsal scales (Figure 1c). The first series of gland pairs (5–7 pairs) started after the parietal scales and extended until the first new mid‐dorsal scale (Figure 1d). The second series started after the new mid‐dorsal scale, in approximately 8–11 pairs separated by the one scale of the new mid‐DSR (Figure 1d). The two mid‐DSR are relatively enlarged, which characterizes the external skin morphology covering the nuchal glands.

The total number of glands in each specimen varied from 12 to 16 pairs and the number was not always symmetrical because the posteriormost gland in one side was absent in four out of the eight individuals. There was no clear difference between sexes (no statistical test was made because of the small sample size). There were no clear relationships between the number of glands and SVL or VEN (Figure 1e,f, Kendall rank correlation test). The size of glands varied, and mean ± SD of length and width of the left glands were 1.17 ± 0.39 (range: 0.48–1.98) mm and 0.68 ± 0.47 (range: 0.33–1.04) mm, respectively. In a single freshly dead specimen with glands containing fluid, the glands' length tended to increase from the first row to the last row (Figure 1g). However, in preserved specimens, the length tended to decrease after the middle gland (Figure 1g). In both freshly dead and preserved specimens, the gland shape tended to change gradually from spherical to elliptical, from the anteriormost to the posteriormost ones (Figure 1h).

### 3.2 Rhabdophis flaviceps

**Figure 2:**
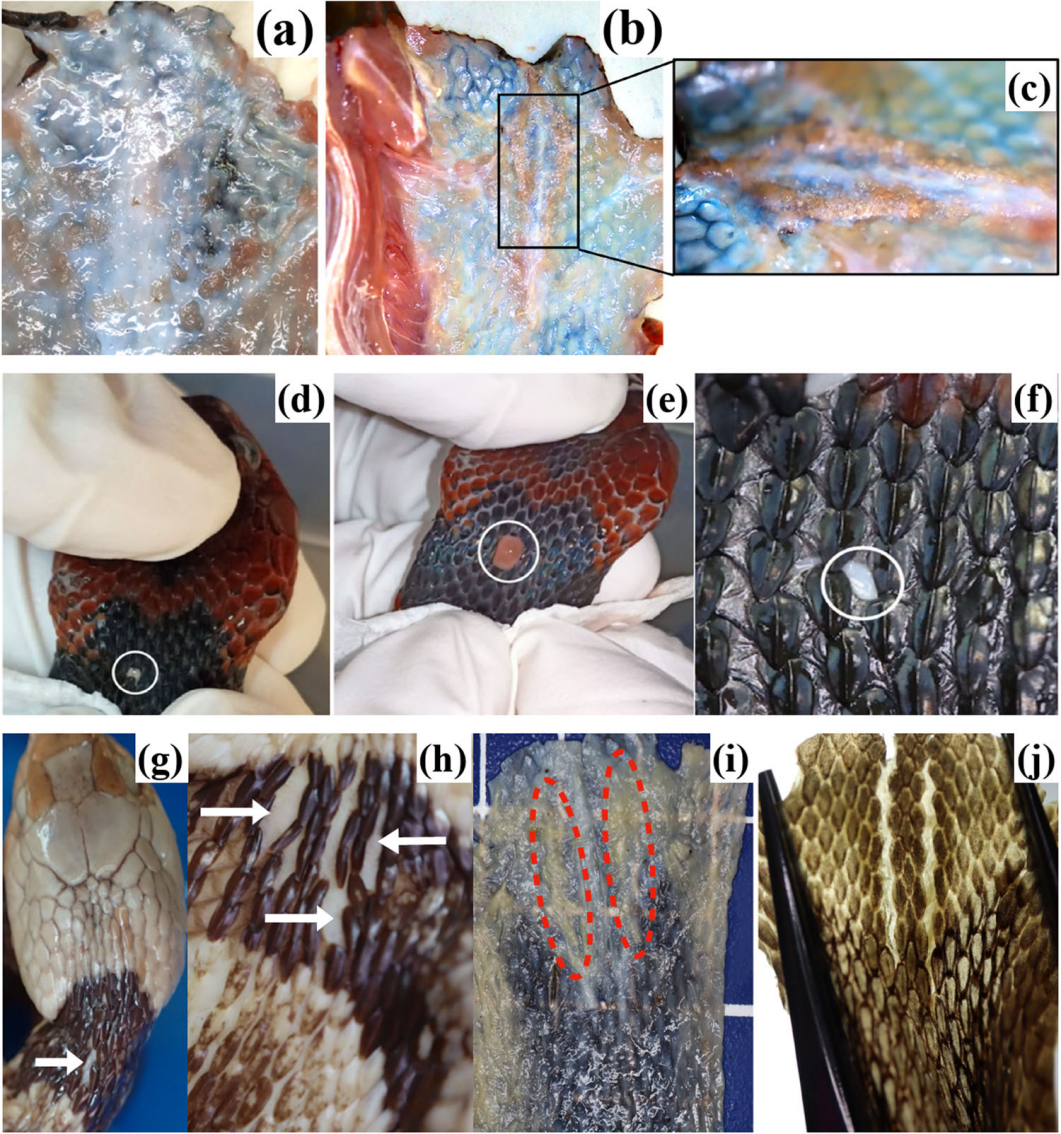
The unsacculated glands of *R. flaviceps* and *R. rhodomelas*. (a) Example of *R. flaviceps* specimen with no trace of glandular structure on the interior side of the neck skin. (b) Example of *R. flaviceps* specimen with putative glandular tissue attached to the underside of the neck skin (black box). (c) Enlargement of the black box in panel b, rotated 90° to the left from the lateral side. It has an unusual structure composed of granular shapes. Fluid can ooze out from the elastic skin; (d) the fluid color was initially milky white (white circle) and (e) gradually turned into reddish (white circle). (f) The observed elastic skin in the place where the fluid oozed out (white circle). (g and h) The elastic skin (indicated with white arrows) observed in several preserved specimens of *R. flaviceps*. (i) Trace of unsacculated glands in a preserved specimen of *R. rhodomelas* (red dotted lines) on the interior of the neck skin. The thin skin is indicated by the visible background. (j) The exterior of the neck skin showing a pair of elastic skin patches in a specimen, which is separated by three middle dorsal scale rows.

Four specimens (1 preserved and 3 freshly dead specimens) showed unusual structures on the underside of the neck skin, whereas seven others did not show any clear structure (Figure 2a–c). The relationship between the occurrence of glands and sex, size, or location was unclear because no statistical tests were performed due to the small sample size representing each group. Among eight freshly dead specimens, only two had an apparent unusual structure in their interior neck skin. Magnification of one of these structures revealed an area composed of many small pinkish granules (Figure 2c). Another specimen had a similar unusual structure, but it was thinner and paler, and no granular shape could be observed (Supporting Information S1: Figures S2 and S3). Both of these unusual structures were located in the interior of the neck skin, starting after the parietal plate, which corresponded to the first ventral and extended to the level of the sixth ventral scale. The maximum length of the structures in these specimens was 13.92 mm and 14.84 mm, respectively. The elongated structures had irregular widths and occurred in symmetrical pairs, separated by the vertebral line. On both sides of the vertebral line, the lateralmost edge of the structure started in the skin area between the middle (0) and first DSR and then extended laterally to the area between the second and third DSR, which approximates the maximum width of the structure.

During fluid extraction from a freshly dead specimen, a white fluid oozed out (Figure 2d), which gradually turned reddish (Figure 2e), possibly mixed with blood. After the dissection of this specimen, we found a small whitish area of skin in the place where the fluid oozed out (Figure 2f). This area exhibits elastic (almost transparent) skin, which was also observed around the neck region in seven of the 11 specimens (with or without the unusual structures) (Figure 2g,h). The relationship of the presence of elastic skin with sex, size, or location was unclear because no statistical tests were performed due to the small sample size representing each group. The position and size of the elastic areas of skin were irregular. The elastic regions were located on the left and/or right sides of the vertebral line and were mostly observed from the mid‐DSR to the fourth DSR. The anterior edge of the elastic skin was situated behind the parietal plate, which corresponded to the first ventral scale, and the elastic skin extended to the level of the fifth ventral scale.

### 3.3 Rhabdophis rhodomelas

The examination of *R. rhodomelas* was conducted only on three preserved specimens. The dissected specimens had thin neck skin, which could be demonstrated by placing the skin on a patterned background, which was visible through the skin (Figure 2i). On the underside of the neck skin, a pair of yellowish elongated tissue structures were found, which were suspected of being remnants of the nuchal glands (Figure 2i). The elastic skin of *R. rhodomelas* was observed in all three specimens by stretching the neck skin. The elastic patches were almost symmetrical to the left and right of the vertebral line and were separated by three mid‐DSR (Figure 2j). The areas of elastic skin started immediately behind the parietal plates and extended for about 10 mm in length, corresponding to the 1st through 4th ventral scales.

### 3.4 *Rhabdophis* spp. of Sulawesi

**Figure 3:**
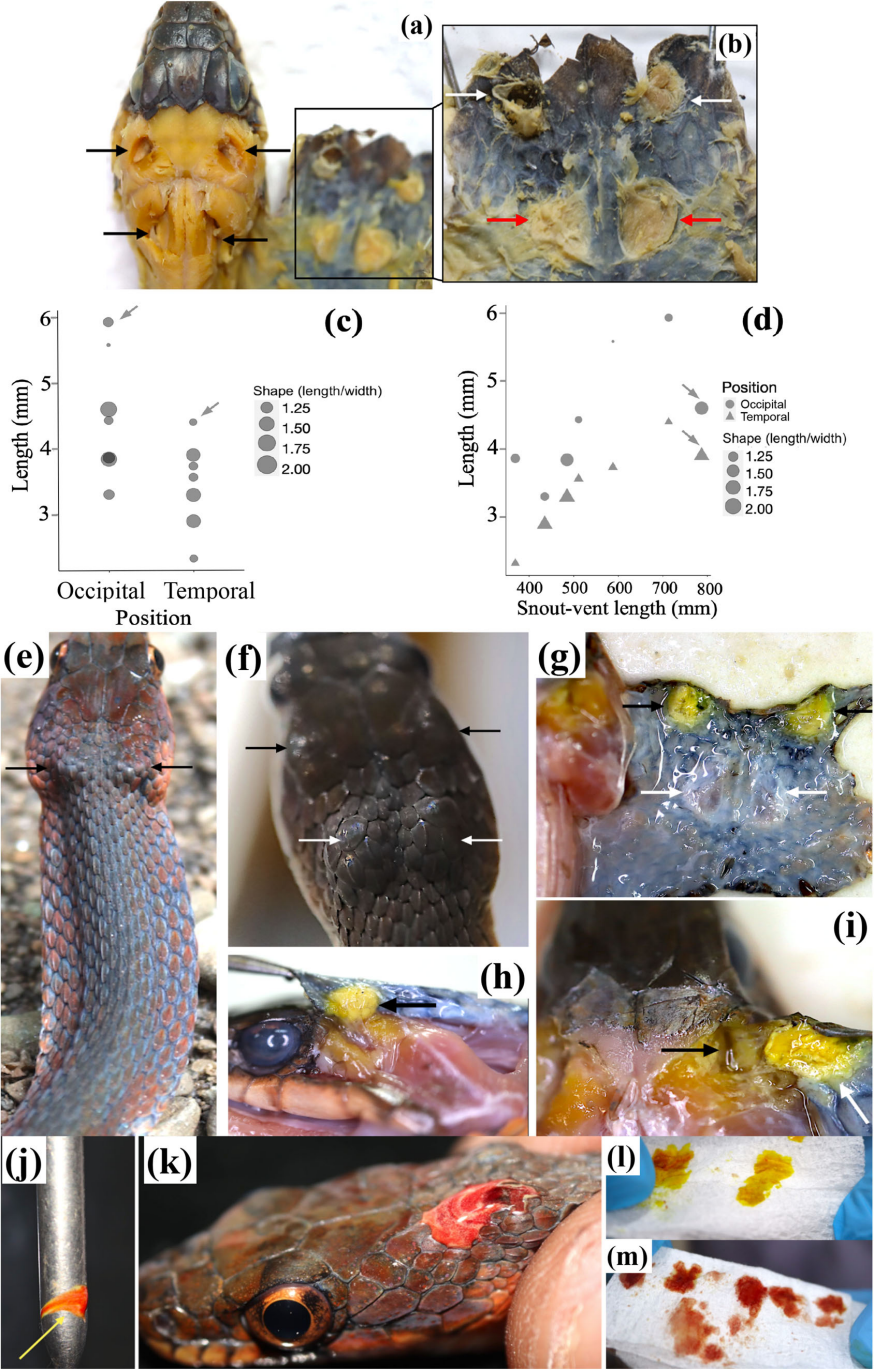
The unusual structure observed in specimens of *Rhabdophis* of Sulawesi. (a) Specimen with dorsal skin of head and neck reflected. On the left, two pairs of pits remain where the temporal and occipital glands had been located (black arrows). (b) Enlargement of the reflected skin, showing the temporal glands attached to the skin of the temporal scales (white arrows) and the occipital glands attached to the anteriormost of skin of the neck (red arrows). (c) Length and shape of occipital and temporal glands (left side) in *Rhabdophis* spp. of Sulawesi. Size of the circle represents shape (length/width). Glands containing fluid are indicated by arrows. (d) Relationship between snout‐vent length (mm) and length of the left temporal glands (triangles) and occipital glands (circles). The size of the symbols indicates their shape (length/width). Glands that contained fluid are indicated by arrows. (e) The neck and anterior regions of body in a live snake, showing the flattened body and round occipital glands (black arrows). (f) Live individual showing two paired swellings in the head (black arrows) and in the anteriormost neck (white arrows). These were the largest such structures observed among all specimens. (g) The temporal glands, attached to the interior of the temporal skin, contain yellowish fluid (black arrows), whereas the occipital glands, attached to the interior of the anteriormost neck skin (white arrows), lack fluid in this specimen, following previous extraction. (h) Lateral view of the left side, showing the temporal gland attached to the temporal skin (black arrow). (i) Dorsal view of the right temporal gland attached to the temporal skin (white arrow), exposing the pit under the parietal plate (black arrow). (j) A mixture of fluids with yellowish and reddish color on a snake hook after it was used to handle the snake. (k) The anteriormost neck skin, showing yellowish and reddish fluid oozing from the occipital gland. (l) First extraction of the occipital glands, showing yellowish fluid absorbed in a Kimwipe. (m) Second extraction of the same gland, showing that most fluid has changed to a reddish color. Photographs a and b are courtesy of Teppei Jono.

All specimens had two symmetrical pairs of unusual structures. The first pair was attached to the underside of the temporal skin (hereafter called temporal glands), while the second pair resides under the occipital skin (hereafter called occipital glands) (Figure 3a,b). There are pits under the temporal glands and parietal plate, which were very obvious compared to other species without these unusual structures. The occipital glands were located on the underside of the occipital skin, approximately 1–2 scales behind the parietal scales, which corresponded to the level of the second and third ventral scales. The pair of occipital glands was symmetrical, and the two glands were separated by approximately three mid‐DSR. Each occipital gland was covered with approximately eight irregularly enlarged scales posterior to the parietal scales.

In the preserved specimens, mean ± SD of length and width of the left temporal gland were 3.27 ± 0.58 (range: 2.32–3.89) mm and 2.26 ± 0.61(range: 1.59–3.09) mm, respectively. Mean ± SD of length and width of the left occipital gland were 4.34 ± 1.35 (range: 3.30–6.98) mm and 3.03 ± 1.36 (range: 1.85–5.50) mm, respectively. The length of the temporal gland was relatively shorter than that of the occipital gland, and the shape of both glands varied from spherical to elliptical (Figure 3c). The glands containing fluid in freshly dead specimens tended to be longer than those in preserved specimens. However, the shape of the gland tended to be spherical when it was filled with fluid (Figure 3c). Temporal gland length tended to increase with increasing SVL (correlation coefficient = 0.886, *p *< 0.001, Pearson correlation test, Figure 3d). Snakes with longer SVL tended to have a longer occipital gland, but no significant correlation was found (correlation coefficient = 0.690, *p *= 0.08, Pearson correlation test, Figure 3d).

In at least three live snakes that we examined, the temporal and occipital glands were evident externally because the glands were swollen with fluid (Figure 3e,f). The swollen structure can be ruptured, oozing, or squirting fluid. Examination of live specimens and extraction of glandular fluid showed that these structures contained a mix of yellowish and reddish fluids (Figure 3g–m). During collection and maintenance, three adult specimens released fluid during handling. One individual squirted the fluid up to approximately one meter when the glandular area was touched with a snake hook.

### 3.5 Bufadienolide Profiles of Snakes

**Figure 4:**
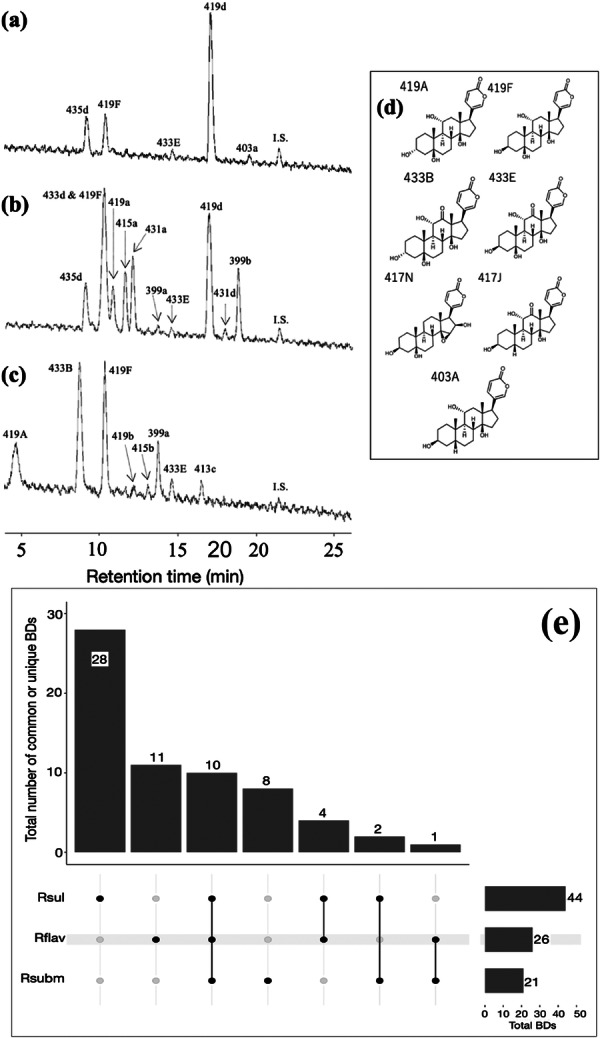
Representative LCMS chromatograms of extracts from the three snake species. (a) *Rhabdophis subminiatus*, (b) *Rhabdophis flaviceps*, (c) *Rhabdophis* of Sulawesi. The X‐axis shows the retention time (min). Total ion chromatogram (TIC) is shown, with tentative names of BDs. IS; internal standard, digitoxigenin. (d) The predicted chemical structures of BDs identified in the three species of snakes. (e) The intersection plot represents the number of detected BDs from the extracted gland fluid of three snake species. Rsul, R flav, and Rsubm represent *Rhabdophis* of Sulawesi, *R. flaviceps*, and *R. subminiatus*, respectively. Total BDs in the lower right corner correspond to the number of BDs detected from species in each line. The Y‐axis shows the number of BDs detected in species indicated with black circles below. The black vertical line shows two or three species that shared common BDs.

LC/MS analyses confirmed that all three species accumulate BDs, although BDs were not detected in 12 out of 16 individuals of *R. subminiatus*. A total of 64 peaks with molecular weight less than 450 were characterized as BDs (Figure 4a–c), and chemical structures of several BDs were estimated (Figure 4d). There were 21, 26, and 44 BDs detected from *R. subminiatus*, *R. flaviceps*, and *Rhabdophis* spp. Sulawesi, respectively (Table 4). The plot in Figure 4e showed the number of common and unique BDs from each species. Among 64 identified BDs, only 17 BDs were common to two or three species, indicating the high variation of BDs in each species. *Rhabdophis* spp. Sulawesi had the highest number of unique BDs, while *R. subminiatus* had the lowest. The chemical structures of the frequently detected compounds such as 419a, 419b, 399b, and 415a from the extracts of all species have not yet been determined (Supporting Information S1: Table S2). Compound 417N was unique to *R. flaviceps*, but was detected in only one of the four specimens examined. Among the ten common BDs identified in all species, 419F, 433E, 419a, 419b, 399b, and 415a were identified in more than 75% of individuals in all species. Compound 433B was common to all species, but it was frequently detected (more than 80%) only in *Rhabdophis* spp. Sulawesi. Of the four compounds shared between *R. flaviceps* and *Rhabdophis* spp. Sulawesi, only 399a was detected in more than 50% individuals. *Rhabdophis flaviceps* and *R. subminiatus* had only one BD in common, 435d, and it was detected in fewer than 50% of individuals.

**Table 4:** Summary of the number of BDs detected from each species.

Species	Total number of BDs		Average of BDs			
	Bufogenin	Bufotoxin	Bufogenin	SD	Bufotoxin	SD
*Rhabdophis subminiatus* (*n* = 4)	21	n.d.	9	7.67	n.d.	n.d.
*Rhabdophis* of Sulawesi (*n* = 6)	44	n.d.	18	10.89	n.d.	n.d.
*Rhabdophis flaviceps* (*n* = 4)	26	n.d.	12	7.80	n.d.	n.d.
*Duttaphrynus melanostictus* (*n* = 11)	71	84	23	8.43	33	9.67
*Phrynoidis aspera* (*n* = 6)	53	52	30	7.86	29	8.57
*Ingerophrynus biporcatus* (*n* = 1)	71	4	71	—	4	—
*Ingerophrynus celebensis* (*n* = 11)	52	66	23	8.93	26	11.05

*Note:* The number of individuals from which BDs were detected is shown in parentheses; n.d. indicates not detected. The total number of BDs showed the number of all compounds identified from the species. The average number of BDs is determined by summing the BDs of each individual and then dividing by the number of individuals. SD indicates standard deviation.

### 3.6 Bufadienolides Profiles of Toads

**Figure 5:**
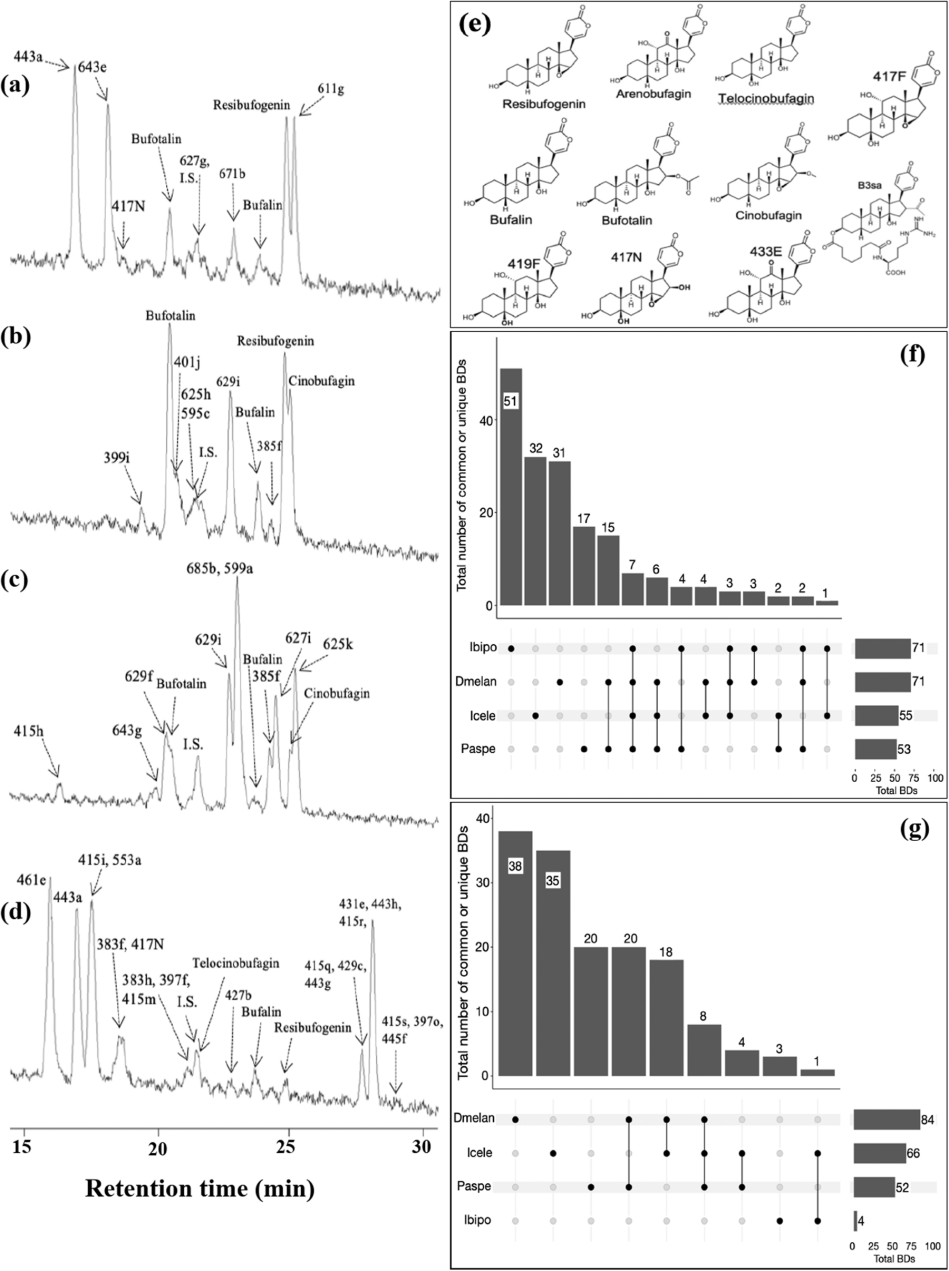
Representative LCMS chromatograms of extracts from the four toad species. (a) *Duttaphrynus melanostictus*, (b) *Phrynoidis aspera*, (c) *Ingerophrynus celebensis*, (d) *I. biporcatus*. X‐axis shows the retention time (min). Total ion chromatogram (TIC) is shown with tentative names of BDs. ﻿IS; internal standard, digitoxigenin. (e) The predicted chemical structure of BDs of bufagenin and bufotoxin (only B3sa) types from the four species. (f) Intersection plot representing the number of detected BDs of bufagenin type from the extract of four species of toads. Ibipo, Dmelan, Icele, Paspe represent *I. biporcatus*, *D. melanostictus*, *I. celebensis,* and *P. aspera*, respectively. Total BDs in the lower right corner correspond to the number of BDs detected from species in each line. Y‐axis shows the number of BDs detected in species indicated with black circles below. The black vertical line shows two or three species that shared common BDs. (g) Intersection plot representing the number of detected BDs of bufotoxin type from the extract of four species of toads. Dmelan, Icele, Paspe, and Ibipo represent *D. melanostictus*, *I. celebensis*, *P. aspera*, and *I. biporcatus*, respectively. Total BDs in the lower right corner correspond to the number of BDs detected from species in each line. Y‐axis shows the number of BDs detected in species indicated with black circles below. The black vertical line shows two or three species that shared common BDs.

Bufadienolides were characterized from all species by 178 peaks of a molecular weight less than 501 (bufogenin type) and 147 peaks of a molecular weight of more than 500 (bufotoxin type) (Figure 5a–d, Supporting Information S1: Tables S3,S4). The predicted chemical structures of some BDs for both bufogenin and bufotoxin types are shown in Figure 5e. Bufogenin‐ and bufotoxin‐type compounds were characterized from all toads in almost equal number, except in *I. biporcatus* (Table 4). The plot shows the number of common and unique compounds of both bufogenin and bufotoxin types identified from all species (Figure 5f,g). A high number of unique bufogenin‐type BDs was detected in each species, indicating extensive variation in BDs. *Ingerophrynus biporcatus* had the highest number of unique bufogenin‐type BDs, and no chemical structure has ever been reported for any of those compounds. Among seven BDs of the bufogenin type that were common to all species, three were identified in more than 90% of individuals (bufalin, resibufogenin, and 403d), and one compound was identified in more than 80% of individuals (401k) (Supporting Information S1: Table S3). *Ingerophrynus biporcatus*, *I. celebensis*, and *D. melanostictus* possessed three common bufogenin‐type BDs, with a moderate detection frequency, in which two of them were estimated to be 419F and 417N. *Ingerophrynus biporcatus* and *I. celebensis* had only one bufogenin type in common, 401f, which was detected in > 90% of individuals. Bufotalin was detected in more than 90% of *D. melanostictus* and *P. asper*, but it was only detected in 25% of *I. celebensis* and was not detected in *I. biporcatus*. There was no bufotoxin common to all of the four toad species. The only bufotoxin with a predicted chemical structure was bufotalin‐3‐suberoyl‐l‐arginine ester (B3sa), which was detected only from *P. asper. Ingerophrynus biporcatus* possessed only four bufotoxin‐type compounds, of which three were unique.

## 4 Discussion

The results of our morphological and chemical analyses confirmed that *R. subminiatus*, *R. flaviceps*, *R. rhodomelas*, and *Rhabdophis* spp. Sulawesi have nuchal glands or similar organs of different types (Table 5). Our observation of the glands of *R. subminiatus* generally agrees with Smith's (1938) description. He observed that the two populations (northern and southern) of *R. subminiatus* exhibit different degrees of development of the nuchal glands. These populations are now recognized as distinct species, with the southern snakes retaining the designation *R. subminiatus* (Liu et al. 2021; David and Vogel 2021). Therefore, the finding of the present study refers specifically to the nuchal glands of *R. subminiatus* sensu stricto. The glandular features of *R. flaviceps* and *R. rhodomelas* generally agree with Smith (1938). He noted that the glands of *R. flaviceps* were not always present and were poorly developed.

**Table 5:** Comparison of gland features among *Rhabdophis* species in Indonesia.

Gland features	*R. subminiatus*	*R. flaviceps*	*Rhabdophis* of Sulawesi
Shape	Chain of spherical‐elliptical	Elongated	Spherical
Number	12–16 pairs	One pair	Two pairs
Location	Neck	Neck	Head
External skin/scale	Enlarged scales	Naked skin	Enlarged and/or irregular scales
Number of BDs	21	26	44

The present study discovered unusual organs in the temporal and occipital areas of *Rhabdophis* spp. of Sulawesi. The type of these organs differed from both the sacculated or unsacculated nuchal glands described by Smith (1938). The organs of *Rhabdophis* spp. Sulawesi have spherical to elliptical shapes, like the sacculated glands, but they are larger in size and are not arranged in chains. In addition, the organs of *Rhabdophis* spp. Sulawesi do not have the elongated form or elastic skin features of the unsacculated glands. Thus, it appears that the form of the organs in Sulawesian *Rhabdophis* represents a novel type. Because the chemical analysis of the fluid from the occipital organs confirmed the presence of BDs, we consider the occipital organs to be comparable to the nuchal glands, and refer to them as occipital glands. Extraction of the occipital glands resulted in a yellowish fluid, often followed by reddish fluid, which may have been blood. Release of blood has been observed in the nuchal gland fluid discharged by other species of *Rhabdophis* (Nakamura 1935). The discharge of blood indicates the presence of blood capillaries associated with the glands (Hutchinson et al. 2007). The temporal organs are similar to the nuchal glands in being attached to the underside of the skin, and they leave pits in the underlying musculature, as often observed by Smith (1938), although he did not specify the species in which that condition occurs. In addition, the dissection of the temporal organs showed that they contain yellowish fluid. Thus, we refer to them as temporal glands. However, the temporal glands were not ruptured, and thus their chemical constituents were not confirmed, and how the fluid is released from them is unclear. Further study is needed to reveal the exact role of the temporal glands and to clarify the occurrence of any different functions between the occipital and temporal glands.

The presence of BDs in the nuchal or occipital glands suggests that *R. subminiatu*s, *R. flaviceps*, and *Rhabdophis* spp. of Sulawesi sequester toxins from toads consumed as food. Bufadienolides were not detected in some individuals of *R. subminiatus*, probably because (1) those snakes had not consumed sufficient toads before they were collected and/or (2) most of the snakes had been used previously in antipredator experiments, which may have caused them to release their toxins. The diversity of BDs in toads and snakes was high, as shown by the high number of unique BDs. All identified BDs in the snakes had a molecular weight of less than 500, indicating the absence of bufotoxins. The chemical structures of most of these unique BDs have not yet been estimated, but several BDs have been reported in previous studies. Compound 417N, which was estimated to be present in *D. melanostictus*, *I. biporcatus*, and *I. celebensis*, has been reported from *R. tigrinus* (Inoue et al. 2021), and 419F, which was estimated to have accumulated in all snake species and three species of toads, has been reported from a toad *Rhinella marina* (Matsukawa et al. 1997). Compound 433E, which was estimated to be present only in *I. celebensis*, has been reported from *R. marina* and from a firefly, *Photinus ignites* (Meng et al. 2016; González et al. 1999). Compound 433B, which was estimated to have accumulated in all snake species, and compound 419A, which was estimated to be present in *Rhabdophis* spp. Sulawesi, have been reported from *R. tigrinus* (Hutchinson et al. 2007).

Bufadienolides with estimated chemical structures may provide hints on the possible biosynthetic pathways. Previous studies showed that, although the BD profiles of toads are reflected in the BDs of *R. tigrinus*, their profiles are not identical to those of the snakes, which is evidence that *R. tigrinus* chemically converts dietary BDs (Inoue et al. 2020, 2021). Many of the BDs identified in *R. subminiatus*, *R. flaviceps*, and *Rhabdophis* spp. Sulawesi were different from the toads' BDs, indicating that under natural conditions, these snakes convert toad‐derived BDs into their distinct profiles of compounds. Nonetheless, there were at least four BDs (433E, 419F, 417N, and 417J) detected from both toads and snakes, suggesting that snakes use some compounds without modification. Bufalin, known as one of the main precursors for BDs of toads and snakes, was detected in more than 90% of all individual toads and in none of the snakes. This suggests that BDs in all toads may be biosynthesized from bufalin through various modifications (Inoue et al. 2020, 2021).

Another important compound characterized in the present study was 419F, which was detected in all species of toads and snakes except for *P. asper*. Compound 419F was detected in more than 80% of individual snakes of all three species. This finding suggests that by feeding on *D. melanostictus*, *I. biporcatus*, or *I. celebensis*, snakes may readily reutilize compound 419F without any chemical conversion. However, snakes may also modify 419F into other potential compounds, as shown in the hypothesized chemical reaction of BDs in *R. tigrinus* (Inoue et al. 2021). Based on the presence of 433E and 433B in all snake species in the present study, we hypothesize the following chemical reaction: oxidation at C‐12 of 419F to produce 433E, followed by epimerization at C‐3 of 433E to produce 433B. The absence of 419F in *P. asper* may explain the low preference of *R. subminiatus* toward this species in tests of prey preference (Anita et al. 2024). Furthermore, the distant phylogenetic relationship between *Phyrnoidis* and the other toad genera (Chan and Grismer 2019) suggests that the capacity for biosynthesizing compound 419F may have been lost in *P. asper*, or it is a new trait developed in the other toad species.

The capacity of toads to synthesize specific compounds may be reflected by some detected BDs. The ability to biosynthesize bufotalin may have been reduced or lost in *Ingerophrynus*, as bufotalin was not found in *I. biporcatus* and was detected in only 3 of 11 *I. celebensis*. Resibufogenin was detected in all toads, but cinobufagin was found only in *P. asper* and *D. melanostictus*. The conversion of resibufogenin into cinobufagin involves acetoxylation of resibufogenin at 16‐C, and this ability may have been lost or never been present in *I. celebensis* and *I. biporcatus*.

The present study detected various BDs of bufotoxin type from toads; however, most of their chemical structures are unknown. The chemical structure of the bufotoxin‐type BDs was estimated only for B3sa, which was detected only from *P. asper*. This indicates that the esterification ability at 3‐OH of bufotalin, which produces B3sa, occurs only in *P. asper*. The low number of bufotoxins identified from *I. biporcatus* may also be related to the high chemosensory preference of *R. subminiatus* toward this toad (Anita et al. 2024). The biodegradation of bufotoxin would involve hydrolytic cleavage of conjugated forms at the C‐3 position (Inoue et al. 2021). Thus, snakes probably would take up BDs more efficiently by feeding on *I. biporcatus*, as no hydrolysis step would be needed to obtain the bufogenins.

This study confirmed that the morphological structures and sequestration of toad toxins are more widespread in *Rhabdophis* species than previously recognized. At least three different types of nuchal glands are present in natricine snakes from Indonesia, including one that is a novel type. These new findings provide further evidence that nuchal glands are more diverse than previously known, particularly in snakes from the southern part of Asia, which is a biogeographically unique region. More research is needed, particularly to understand the environmental and evolutionary pressures that may drive this diversity.

## Author Contributions

**Syahfitri Anita:** conceptualization, methodology, data curation, investigation, validation, project administration, visualization, funding acquisition, writing – original draft, writing – review and editing, formal analysis, resources. **Takato Inoue:** investigation, methodology, validation, formal analysis, data curation, writing – review and editing. **Aya Inoue:** methodology, investigation, validation, formal analysis, data curation, writing – review and editing. **Koshiro Eto:** methodology, investigation, writing – review and editing. **Amir Hamidy:** conceptualization, writing – review and editing, project administration, resources, data curation, methodology. **Naoki Mori:** methodology, investigation, formal analysis, writing – review and editing, project administration, supervision, data curation. **Akira Mori:** conceptualization, methodology, data curation, investigation, validation, formal analysis, supervision, funding acquisition, visualization, project administration, resources, writing – original draft, writing – review and editing.

## Ethics Statement

The experimental procedures were approved by the Committee of Ethical Clearance for Research of the Indonesian Institute of Sciences (Letter number: 9/klirens/XI/2020).

## Conflicts of Interest

The authors declare no conflicts of interest.

## 1 Peer Review

The peer review history for this article is available at https://www.webofscience.com/api/gateway/wos/peer-review/10.1002/jmor.70071.

## Supporting information

Supplementary.

## Data Availability

The data that support the findings of this study are openly available in figshare at https://figshare.com/s/1eeb7a1d1102f8224fd2.
