# Supplementary material for: Morphology and Chemical Composition of the Nuchal Glands of Indonesian Snakes With a Description of a Novel Type of Glands
Source: J Morphol. 2025 Aug 14;286(8):e70071. doi: 10.1002/jmor.70071 (PMC12352018; doi:10.1002/jmor.70071)
Supplement: Supplementary file 1 — Supplementary. [file JMOR-286-e70071-s001.docx]

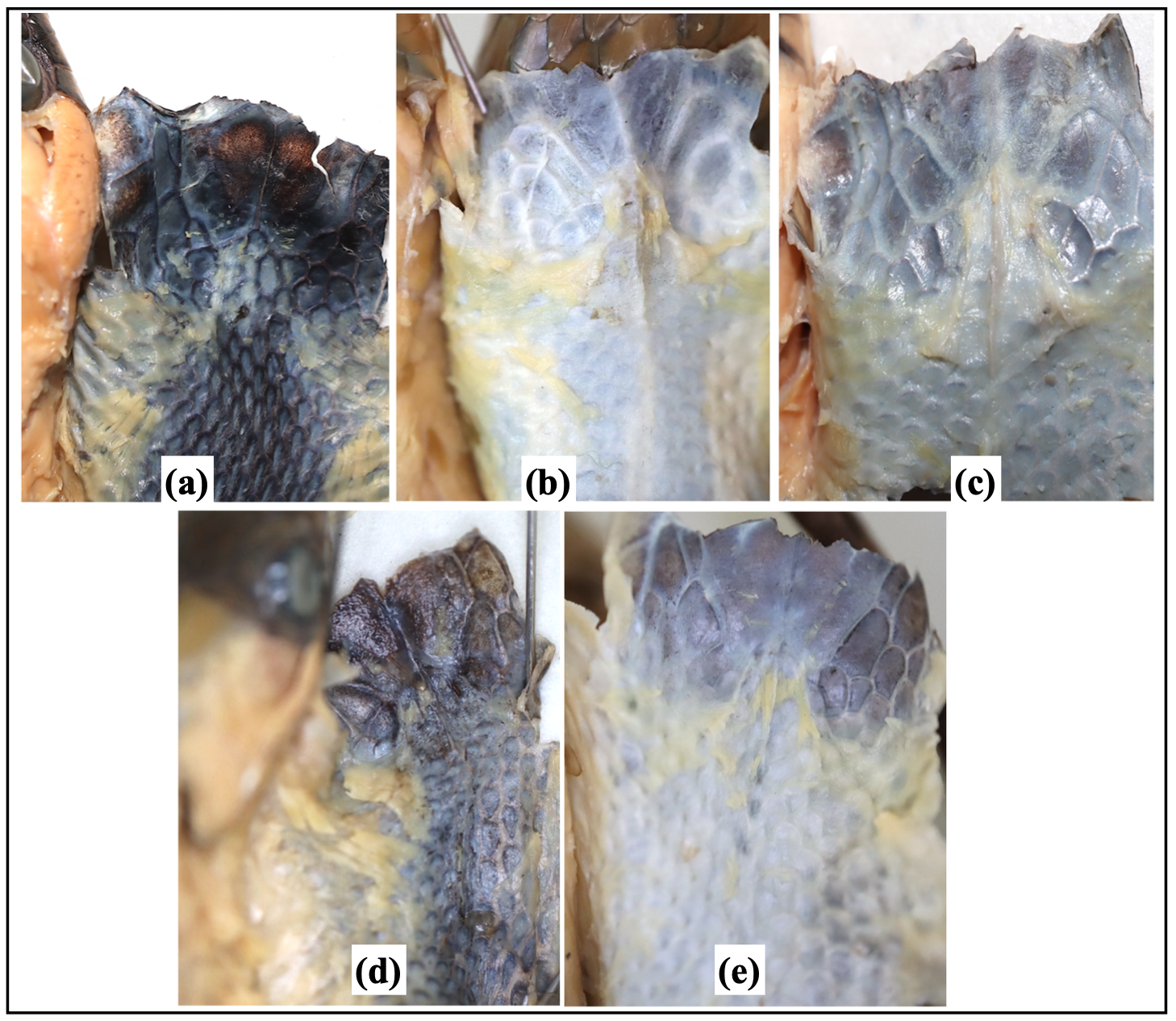


**Figure S1**. Examples of neck skin without trace of nuchal glands in (a) *Rhabdophis chrysargos*, (b) *Fowlea melanzostus,* (c) *Xenochrophis* *trianguligerus*, (d) *Tropidonophis mairii* and (e) *Tropidonophis doriae.*

**
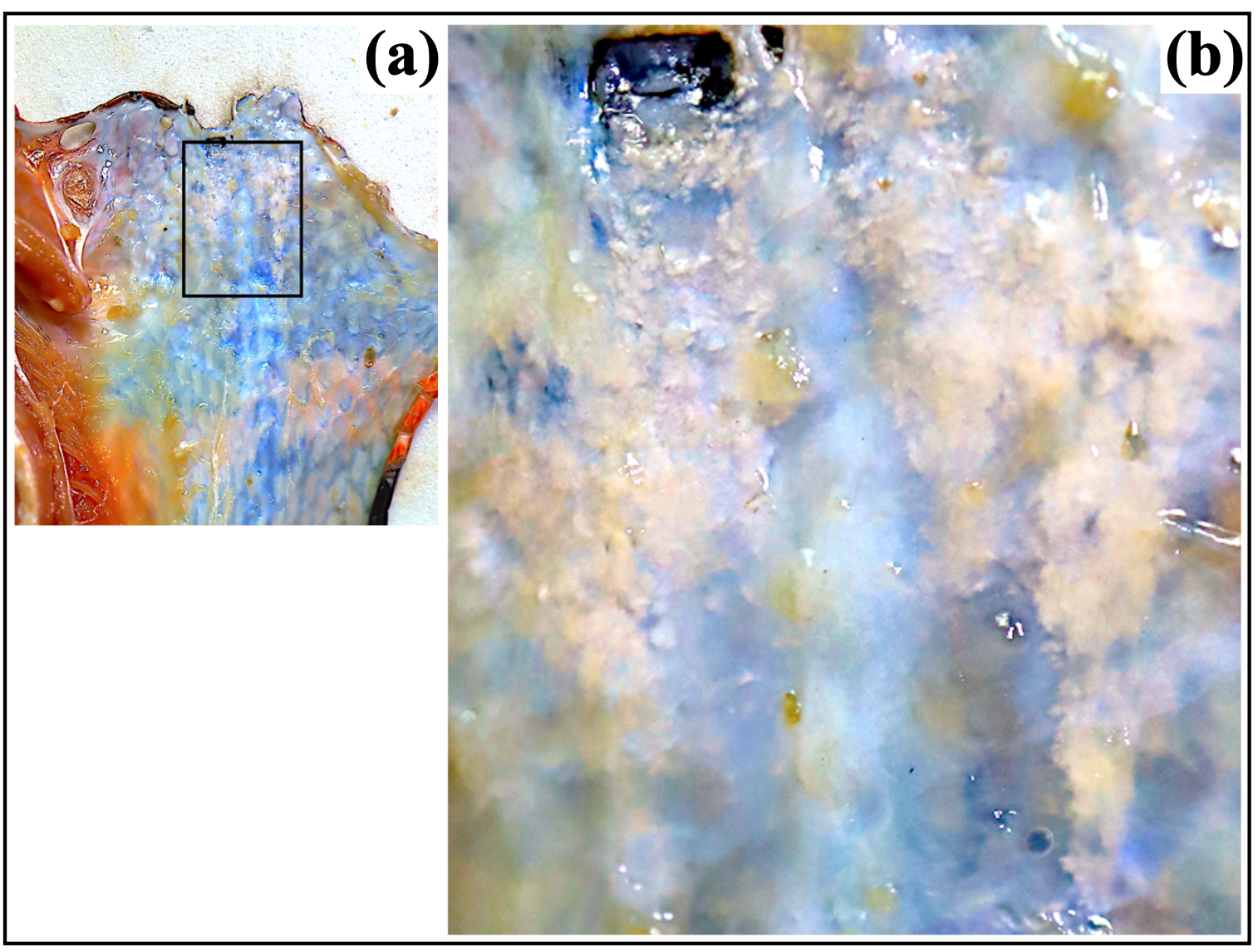
**

**Figure S2**. (a) A specimen of *R. flaviceps* having a unusual and thin structure in the interior side of the neck skin (black box). (b) Enlargement of the black box showing that this unusual structure resembles the form of unsacculated nuchal glands reported by Smith (1938).


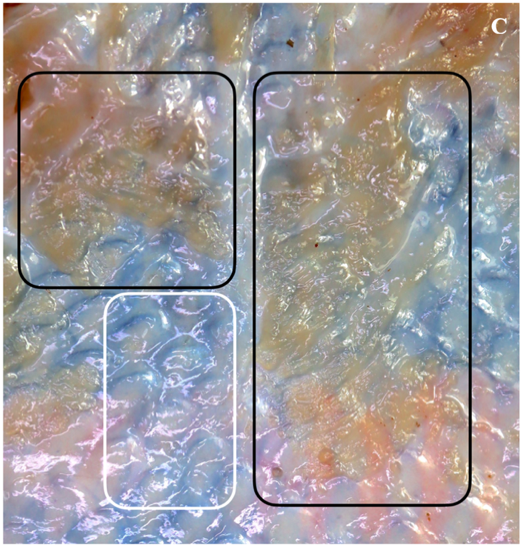

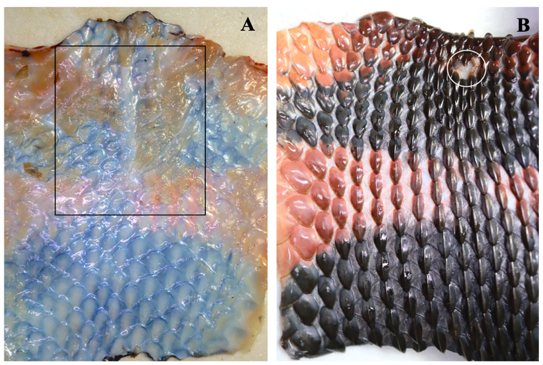


**Figure S3**. Left, the black box shows the remnants of paired, unsacculated glands following fluid extraction of *R. flaviceps* with identified 15 BDs. Right, the enlarged section of the area inside the black box in the left image, showing areas with traces of tissue (black boxes) that possibly are nuchal glands and a clear area without tissue or a trace of nuchal gland (white box).

**Table S1**. Morphological characters used to identify species. DSR, dorsal scale rows at midbody; VEN, number of ventrals; SC, number of subcaudals; SL, number of supralabials.

| Species | DSR | VEN | SC | SL | References |
| --- | --- | --- | --- | --- | --- |
| *Rhabdophis subminiatus* | 19 | 144–184 | 56–97 | 7–9 | Schlegel, 1837; Lang, 2017 |
| *Rhabdophis* of Sulawesi | 21 | 154–161 | 64–79 | 9 | Günther, 1858; Lang & Vogel, 2005 |
| *Rhabdophis callistus* | 21 | 156 | 76 | 8–9 | Günther, 1873; Lang & Vogel, 2005 |
| *Rhabdophis chrysargos* | 19 | 143–175 | 51–89 | 8–9 | Schlegel, 1837; Lang, 2017 |
| *Rhabdophis flaviceps* | 19 | 120–138 | 49–60 | 8 | Duméril, Bibron & Duméril, 1854; Das, 2018 |
| *Rhabdophis rhodomelas* | 19 | 126–136 | 41–56 | - | Boie, 1827; Das, 2018 |
| *Xenochrophis trianguligerus* | 19 | 130–145 | 67–99 | 8–10 | Boie, 1827; Lang, 2017 |
| *Fowlea melanzostus* | 19 | 127–152 | 48–80 | 9 | Gravenhorst, 1807; Lang, 2017 |
| *Tropidonophis mairii* | 15 | 140–166 | 58–76 | 8 | Gray, 1842; Malnate & Underwood, 1988 |
| *Tropidonophis doriae* | 17 | 134–159 | 71–90 | 8 | Boulenger, 1898; Malnate & Underwood, 1988 |

Boie, F. (1827). Bemerkungen über Merrem’s Versuch eines Systems der Amphibien, 1. Lieferung: Ophidier. *Isis von Oken*, *20*, 508–566.

Boulenger, G. A. (1898). An account of the reptiles and batrachians collected by Dr. L. Loria in British New Guinea. *Annali Del Museo Civico Di Storia Naturale Di Genova*, *18*, 694–710.

Duméril, A. M. C., Bibron, G., & Duméril, A. H. A. (1854). Erpétologie générale ou Histoire Naturelle complète des Reptiles. *Paris, France: Ome Septième*, *7*.

Gravenhorst, J. L. C. (1807). *Vergleichende Übersicht des Linneischen und einiger neueren zoologischen Systeme, nebst dem eingeschalteten Verzeichnisse der zoologischen Sammlung des Verfassers und den Beschreibungen neuer Thierarten, die in derselben vorhanden sind. Heinrich Dietrich* (pp. i–xvi + 1-476).

Gray, J. E. (1842). Description of two hitherto unrecorded species of reptiles from New Zealand; presented to the British Museum by Dr. Dieffenbach. *Zoological Miscellany*, *2*(72).1-72.

Günther, A. (1858). *Catalogue of Colubrine Snakes of the British Museum*, *I–XVI*, 1–281.

Günther, A. (1873). Notes on some reptiles and batrachians obtained by Dr. Bernhard Meyer in Celebes and the Philippine Islands. *Proceedings of the Zoological Society*,165–172.

Malnate, E. V., & Underwood, G. (1988). Australasian natricine snakes of the genus *Tropidonophis*. *Proceedings of the Academy of Natural Sciences of Philadelphia*, *140*(1), 59–201.

Schlegel, H. (1837). Essai sur la Physionomie des Serpens. Partie Descriptive. *La Haye (J. Kips, J. HZ. Et W. P. Van Stockum)*, *606 S. + xvi*.

**Table S2**. Detection rate of each BD (bufogenin) characterized from snakes in the present study. Numbers in the table are percentages of individuals containing that compound.

| No | Compound name | *R. subminiatus* (*n* = 4) | *R. flaviceps* (*n* = 4) | *R. chryargoides* (*n* = 6) |
| --- | --- | --- | --- | --- |
| 1 | 419F | 100 | 75 | 83 |
| 2 | 433E | 75 | 100 | 83 |
| 3 | 419a | 50 | 75 | 83 |
| 4 | 419b | 50 | 75 | 83 |
| 5 | 399b | 75 | 50 | 67 |
| 6 | 415a | 50 | 75 | 50 |
| 7 | 433B | 25 | 25 | 83 |
| 8 | 419d | 75 | 50 | 17 |
| 9 | 431d | 50 | 25 | 33 |
| 10 | 417g | 25 | 50 | 17 |
| 11 | 403a | 75 | 0 | 17 |
| 12 | 417f | 25 | 0 | 50 |
| 13 | 435d | 50 | 25 | 0 |
| 14 | 399a | 0 | 50 | 67 |
| 15 | 433f | 0 | 25 | 50 |
| 16 | 417d | 0 | 50 | 17 |
| 17 | 435a | 0 | 25 | 33 |
| 18 | 435b | 25 | 0 | 0 |
| 19 | 447a | 25 | 0 | 0 |
| 20 | 403A | 25 | 0 | 0 |
| 21 | 417J | 25 | 0 | 0 |
| 22 | 431b | 25 | 0 | 0 |
| 23 | 431c | 25 | 0 | 0 |
| 24 | 435i | 25 | 0 | 0 |
| 25 | 419e | 25 | 0 | 0 |
| 26 | 433d | 0 | 100 | 0 |
| 27 | 433c | 0 | 75 | 0 |
| 28 | 431a | 0 | 75 | 0 |
| 29 | 433a | 0 | 25 | 0 |
| 30 | 413a | 0 | 25 | 0 |
| 31 | 435h | 0 | 25 | 0 |
| 32 | 401a | 0 | 25 | 0 |
| 33 | 417N | 0 | 25 | 0 |
| 34 | 401b | 0 | 25 | 0 |
| 35 | 401c | 0 | 25 | 0 |
| 36 | 403b | 0 | 25 | 0 |
| 37 | 435e | 0 | 0 | 83 |
| 38 | 415b | 0 | 0 | 83 |
| 39 | 413c | 0 | 0 | 83 |
| 40 | 433g | 0 | 0 | 67 |
| 41 | 417c | 0 | 0 | 50 |
| 42 | 433h | 0 | 0 | 50 |
| 43 | 429b | 0 | 0 | 50 |
| 44 | 427c | 0 | 0 | 50 |
| 45 | 449b | 0 | 0 | 33 |
| 46 | 433b | 0 | 0 | 33 |
| 47 | 417a | 0 | 0 | 33 |
| 48 | 417b | 0 | 0 | 33 |
| 49 | 427a | 0 | 0 | 33 |
| 50 | 427b | 0 | 0 | 33 |
| 51 | 449a | 0 | 0 | 17 |
| 52 | 419A | 0 | 0 | 17 |
| 53 | 435c | 0 | 0 | 17 |
| 54 | 435f | 0 | 0 | 17 |
| 55 | 433e | 0 | 0 | 17 |
| 56 | 435g | 0 | 0 | 17 |
| 57 | 413b | 0 | 0 | 17 |
| 58 | 449c | 0 | 0 | 17 |
| 59 | 449d | 0 | 0 | 17 |
| 60 | 419c | 0 | 0 | 17 |
| 61 | 429a | 0 | 0 | 17 |
| 62 | 417e | 0 | 0 | 17 |
| 63 | 433i | 0 | 0 | 17 |
| 64 | 415c | 0 | 0 | 17 |

**Table S3.** Detection rate of each BD of bufogenin type characterized from toads in the present study. Numbers in table are percentages of individuals containing that compound.

| No | BDs name | *D. melanostictus*  (*n* = 11) | *P. aspera*  (*n* = 6) | *I. celebensis* (*n* = 11) | *I. biporcatus*  (*n* = 1) |
| --- | --- | --- | --- | --- | --- |
| 1 | 415a | 17 | 0 | 0 | 0 |
| 2 | 435a | 0 | 0 | 0 | 100 |
| 3 | 401a | 17 | 0 | 0 | 0 |
| 4 | 415b | 17 | 0 | 0 | 0 |
| 5 | 457a | 0 | 0 | 0 | 100 |
| 6 | 387a | 0 | 0 | 0 | 100 |
| 7 | 435b | 0 | 0 | 0 | 100 |
| 8 | 435c | 0 | 0 | 18 | 0 |
| 9 | 475a | 17 | 0 | 0 | 0 |
| 10 | 433a | 8 | 0 | 0 | 0 |
| 11 | 419a | 8 | 0 | 9 | 0 |
| 12 | 383a | 0 | 0 | 9 | 0 |
| 13 | 419F | 25 | 0 | 100 | 100 |
| 14 | 415c | 25 | 0 | 0 | 0 |
| 15 | 433b | 42 | 33 | 9 | 0 |
| 16 | 461a | 0 | 17 | 0 | 0 |
| 17 | 433c | 17 | 0 | 0 | 0 |
| 18 | 415d | 8 | 0 | 0 | 0 |
| 19 | 475b | 17 | 17 | 0 | 0 |
| 20 | 419b | 0 | 17 | 0 | 0 |
| 21 | 475c | 75 | 100 | 36 | 0 |
| 22 | 403a | 8 | 50 | 27 | 0 |
| 23 | 399a | 17 | 0 | 0 | 0 |
| 24 | 417a | 0 | 0 | 64 | 0 |
| 25 | 397a | 0 | 0 | 64 | 0 |
| 26 | 397b | 25 | 0 | 9 | 0 |
| 27 | 415e | 0 | 17 | 0 | 0 |
| 28 | 419c | 0 | 0 | 9 | 0 |
| 29 | 433d | 0 | 0 | 82 | 0 |
| 30 | 461b | 0 | 0 | 73 | 0 |
| 31 | 417F | 17 | 0 | 0 | 0 |
| 32 | 401b | 8 | 0 | 0 | 0 |
| 33 | 457b | 0 | 0 | 0 | 100 |
| 34 | 401c | 17 | 0 | 0 | 0 |
| 35 | 461c | 25 | 0 | 0 | 0 |
| 36 | 399b | 25 | 0 | 0 | 0 |
| 37 | 383b | 8 | 0 | 0 | 0 |
| 38 | 433E | 0 | 0 | 18 | 0 |
| 39 | 413a | 17 | 0 | 0 | 0 |
| 40 | 445a | 17 | 0 | 0 | 0 |
| 41 | 417b | 8 | 0 | 0 | 0 |
| 42 | 399c | 17 | 0 | 0 | 0 |
| 43 | 431a | 0 | 17 | 0 | 0 |
| 44 | 417c | 0 | 0 | 18 | 0 |
| 45 | 415f | 17 | 33 | 0 | 0 |
| 46 | 459a | 17 | 0 | 0 | 0 |
| 47 | 417d | 17 | 0 | 0 | 0 |
| 48 | 383c | 17 | 0 | 0 | 0 |
| 49 | 397c | 0 | 0 | 0 | 100 |
| 50 | 461d | 8 | 0 | 0 | 0 |
| 51 | 403b | 0 | 0 | 18 | 0 |
| 52 | 417e | 33 | 17 | 0 | 0 |
| 53 | 459b | 42 | 17 | 0 | 100 |
| 54 | 399d | 17 | 0 | 0 | 0 |
| 55 | 401d | 25 | 100 | 0 | 0 |
| 56 | 461e | 0 | 0 | 0 | 100 |
| 57 | 415g | 25 | 0 | 0 | 100 |
| 58 | 433e | 0 | 17 | 0 | 0 |
| 59 | 459c | 8 | 0 | 27 | 0 |
| 60 | 419d | 17 | 0 | 0 | 0 |
| 61 | 473a | 0 | 0 | 9 | 0 |
| 62 | 461f | 0 | 0 | 0 | 100 |
| 63 | 415h | 0 | 0 | 73 | 0 |
| 64 | Arenobufagin | 75 | 50 | 100 | 0 |
| 65 | 399e | 0 | 50 | 0 | 0 |
| 66 | 417f | 0 | 17 | 0 | 0 |
| 67 | 431b | 0 | 17 | 0 | 0 |
| 68 | 459d | 0 | 0 | 27 | 0 |
| 69 | 443a | 58 | 0 | 0 | 100 |
| 70 | 417g | 83 | 0 | 9 | 100 |
| 71 | 419e | 83 | 0 | 0 | 100 |
| 72 | 383d | 0 | 0 | 0 | 100 |
| 73 | 397d | 8 | 33 | 0 | 0 |
| 74 | 459e | 0 | 0 | 27 | 0 |
| 75 | 415i | 0 | 0 | 0 | 100 |
| 76 | 383e | 0 | 17 | 0 | 100 |
| 77 | 401e | 8 | 67 | 0 | 0 |
| 78 | 417h | 0 | 0 | 45 | 0 |
| 79 | 431c | 0 | 0 | 0 | 100 |
| 80 | 399f | 0 | 83 | 0 | 0 |
| 81 | 445b | 0 | 0 | 0 | 100 |
| 82 | 401f | 0 | 0 | 91 | 100 |
| 83 | 417i | 0 | 0 | 0 | 100 |
| 84 | 461g | 8 | 17 | 0 | 0 |
| 85 | 383f | 8 | 67 | 0 | 100 |
| 86 | 399g | 0 | 67 | 0 | 0 |
| 87 | 461h | 0 | 17 | 0 | 0 |
| 88 | 415j | 0 | 67 | 0 | 0 |
| 89 | 417N | 58 | 0 | 55 | 100 |
| 90 | 397e | 0 | 17 | 45 | 0 |
| 91 | 399h | 0 | 0 | 82 | 0 |
| 92 | 459f | 0 | 0 | 100 | 0 |
| 93 | 403c | 8 | 83 | 0 | 0 |
| 94 | 401g | 0 | 50 | 0 | 100 |
| 95 | 419f | 0 | 0 | 0 | 100 |
| 96 | 415k | 17 | 33 | 0 | 0 |
| 97 | 445c | 0 | 0 | 0 | 100 |
| 98 | 443b | 0 | 0 | 18 | 0 |
| 99 | 399i | 8 | 100 | 9 | 100 |
| 100 | 401h | 75 | 67 | 100 | 100 |
| 101 | 383g | 0 | 0 | 0 | 100 |
| 102 | 403d | 92 | 100 | 100 | 100 |
| 103 | 415l | 0 | 0 | 27 | 0 |
| 104 | 443c | 0 | 0 | 18 | 0 |
| 105 | 431d | 17 | 0 | 0 | 0 |
| 106 | 385a | 17 | 0 | 0 | 0 |
| 107 | 399j | 0 | 33 | 0 | 0 |
| 108 | 445d | 0 | 0 | 55 | 0 |
| 109 | 403e | 0 | 0 | 18 | 0 |
| 110 | 401i | 0 | 0 | 9 | 0 |
| 111 | 399k | 0 | 0 | 9 | 0 |
| 112 | Bufotalin | 92 | 100 | 27 | 0 |
| 113 | 401j | 0 | 50 | 0 | 100 |
| 114 | 383h | 0 | 0 | 0 | 100 |
| 115 | 397f | 0 | 0 | 0 | 100 |
| 116 | 415m | 0 | 0 | 0 | 100 |
| 117 | 459g | 25 | 0 | 0 | 0 |
| 118 | 401k | 92 | 83 | 91 | 100 |
| 119 | 427a | 67 | 0 | 27 | 0 |
| 120 | 415n | 50 | 0 | 0 | 0 |
| 121 | 429a | 0 | 0 | 0 | 100 |
| 122 | 399l | 0 | 67 | 0 | 0 |
| 123 | 385b | 0 | 50 | 0 | 0 |
| 124 | 383i | 8 | 33 | 0 | 0 |
| 125 | 399m | 33 | 100 | 0 | 0 |
| 126 | Telocinobufagin | 92 | 67 | 27 | 100 |
| 127 | 429b | 0 | 0 | 0 | 100 |
| 128 | 385c | 0 | 0 | 0 | 100 |
| 129 | 401l | 0 | 0 | 9 | 0 |
| 130 | 397g | 0 | 0 | 0 | 100 |
| 131 | 415o | 0 | 0 | 0 | 100 |
| 132 | 397h | 0 | 0 | 0 | 100 |
| 133 | 401m | 17 | 83 | 0 | 0 |
| 134 | 401n | 0 | 0 | 18 | 0 |
| 135 | 397i | 0 | 0 | 0 | 100 |
| 136 | 403f | 8 | 100 | 9 | 0 |
| 137 | 427b | 0 | 0 | 0 | 100 |
| 138 | 385d | 0 | 100 | 0 | 0 |
| 139 | 397j | 0 | 0 | 45 | 0 |
| 140 | 443d | 0 | 0 | 0 | 100 |
| 141 | 385e | 0 | 0 | 9 | 0 |
| 142 | 401o | 0 | 0 | 82 | 0 |
| 143 | 415p | 0 | 0 | 0 | 100 |
| 144 | Bufalin | 92 | 100 | 100 | 100 |
| 145 | 401p | 33 | 0 | 0 | 0 |
| 146 | 397k | 0 | 0 | 0 | 100 |
| 147 | 399n | 0 | 0 | 0 | 100 |
| 148 | 417j | 0 | 0 | 0 | 100 |
| 149 | 385f | 0 | 100 | 73 | 0 |
| 150 | 445e | 0 | 0 | 9 | 0 |
| 151 | 383j | 8 | 100 | 0 | 0 |
| 152 | Resibufogenin | 92 | 100 | 100 | 100 |
| 153 | 427c | 0 | 0 | 36 | 0 |
| 154 | 399o | 8 | 33 | 0 | 0 |
| 155 | Cinobufagin | 8 | 100 | 0 | 0 |
| 156 | 385g | 0 | 100 | 0 | 100 |
| 157 | 443e | 0 | 17 | 0 | 0 |
| 158 | 383k | 8 | 0 | 0 | 0 |
| 159 | 385h | 0 | 0 | 0 | 100 |
| 160 | 397l | 0 | 0 | 0 | 100 |
| 161 | 397m | 0 | 0 | 0 | 100 |
| 162 | 397n | 0 | 0 | 0 | 100 |
| 163 | 443f | 0 | 0 | 0 | 100 |
| 164 | 415q | 0 | 0 | 0 | 100 |
| 165 | 429c | 0 | 0 | 0 | 100 |
| 166 | 443g | 0 | 0 | 0 | 100 |
| 167 | 385i | 0 | 0 | 18 | 0 |
| 168 | 431e | 0 | 0 | 0 | 100 |
| 169 | 443h | 0 | 0 | 0 | 100 |
| 170 | 415r | 0 | 0 | 0 | 100 |
| 171 | 415s | 0 | 0 | 0 | 100 |
| 172 | 397o | 0 | 0 | 0 | 100 |
| 173 | 445f | 0 | 0 | 0 | 100 |
| 174 | 383l | 0 | 0 | 0 | 100 |
| 175 | 401q | 0 | 0 | 0 | 100 |
| 176 | 443i | 0 | 0 | 0 | 100 |
| 177 | 397p | 0 | 0 | 0 | 100 |
| 178 | 397q | 0 | 0 | 0 | 100 |

**Table S4**. Detection rate of each BD of bufotoxin type characterized from toads in the present study. Numbers in table are percentages of individuals containing that compound.

| No | BDs name | *D. melanostictus* (*n* = 11) | *P. aspera*  (*n* = 6) | *I. celebensis*  (*n* = 11) | *I. biporcatus*  (*n* = 1) |
| --- | --- | --- | --- | --- | --- |
| 1 | 613f | 100 | 100 | 100 | 0 |
| 2 | 611g | 91 | 100 | 100 | 0 |
| 3 | 627d | 91 | 50 | 91 | 0 |
| 4 | 629f | 91 | 0 | 100 | 0 |
| 5 | 671b | 100 | 100 | 27 | 0 |
| 6 | 597a | 36 | 100 | 82 | 0 |
| 7 | 615a | 64 | 0 | 91 | 0 |
| 8 | 643g | 55 | 0 | 100 | 0 |
| 9 | 625k | 18 | 50 | 100 | 0 |
| 10 | 643d | 91 | 50 | 18 | 0 |
| 11 | 599a | 64 | 0 | 73 | 0 |
| 12 | 643h | 27 | 0 | 100 | 0 |
| 13 | 625e | 91 | 50 | 0 | 0 |
| 14 | 627c | 18 | 0 | 100 | 0 |
| 15 | 625d | 82 | 50 | 0 | 0 |
| 16 | 629g | 91 | 0 | 18 | 0 |
| 17 | 627i | 18 | 0 | 91 | 0 |
| 18 | 645c | 91 | 0 | 9 | 0 |
| 19 | 613b | 82 | 17 | 9 | 0 |
| 20 | 641d | 0 | 0 | 100 | 0 |
| 21 | 657f | 0 | 0 | 100 | 0 |
| 22 | 657g | 82 | 0 | 18 | 0 |
| 23 | 625b | 91 | 0 | 0 | 0 |
| 24 | 643e | 91 | 0 | 0 | 0 |
| 25 | 701b | 73 | 0 | 18 | 0 |
| 26 | 629e | 0 | 0 | 91 | 0 |
| 27 | 627e | 64 | 0 | 27 | 0 |
| 28 | 625h | 45 | 83 | 0 | 0 |
| 29 | 627h | 18 | 0 | 73 | 0 |
| 30 | 629b | 82 | 0 | 0 | 0 |
| 31 | 625c | 82 | 0 | 0 | 0 |
| 32 | 643c | 82 | 0 | 0 | 0 |
| 33 | 609b | 27 | 100 | 0 | 0 |
| 34 | 609e | 27 | 100 | 0 | 0 |
| 35 | 613e | 0 | 0 | 82 | 0 |
| 36 | 643b | 55 | 0 | 18 | 0 |
| 37 | 627b | 0 | 0 | 73 | 0 |
| 38 | 643f | 0 | 0 | 73 | 0 |
| 39 | 627f | 18 | 100 | 0 | 0 |
| 40 | 611a | 64 | 0 | 0 | 0 |
| 41 | 629c | 0 | 0 | 64 | 0 |
| 42 | 615b | 64 | 0 | 0 | 0 |
| 43 | 641e | 0 | 0 | 64 | 0 |
| 44 | 591a | 9 | 100 | 0 | 0 |
| 45 | 609f | 9 | 100 | 0 | 0 |
| 46 | 611f | 0 | 0 | 64 | 0 |
| 47 | 609g | 9 | 100 | 0 | 0 |
| 48 | 711a | 9 | 100 | 0 | 0 |
| 49 | 641a | 55 | 0 | 0 | 0 |
| 50 | 659b | 27 | 0 | 27 | 0 |
| 51 | 609a | 55 | 0 | 0 | 0 |
| 52 | 613a | 0 | 0 | 55 | 0 |
| 53 | 613c | 55 | 0 | 0 | 0 |
| 54 | 625f | 45 | 17 | 0 | 0 |
| 55 | 609c | 0 | 100 | 0 | 0 |
| 56 | 609d | 0 | 100 | 0 | 0 |
| 57 | 625i | 9 | 83 | 0 | 0 |
| 58 | 685b | 0 | 50 | 27 | 0 |
| 59 | 697c | 0 | 100 | 0 | 0 |
| 60 | 629i | 0 | 67 | 9 | 0 |
| 61 | 641i | 0 | 0 | 45 | 0 |
| 62 | 771a | 9 | 67 | 0 | 0 |
| 63 | 615c | 45 | 0 | 0 | 0 |
| 64 | 687a | 27 | 0 | 9 | 0 |
| 65 | 627a | 36 | 0 | 0 | 0 |
| 66 | 641c | 36 | 0 | 0 | 0 |
| 67 | 657d | 36 | 0 | 0 | 0 |
| 68 | 611d | 0 | 67 | 0 | 0 |
| 69 | 641f | 36 | 0 | 0 | 0 |
| 70 | 627g | 18 | 0 | 18 | 0 |
| 71 | B3sa | 0 | 67 | 0 | 0 |
| 72 | 697b | 9 | 50 | 0 | 0 |
| 73 | 713a | 9 | 50 | 0 | 0 |
| 74 | 669a | 0 | 67 | 0 | 0 |
| 75 | 575a | 27 | 0 | 0 | 0 |
| 76 | 639a | 27 | 0 | 0 | 0 |
| 77 | 657a | 27 | 0 | 0 | 0 |
| 78 | 629a | 27 | 0 | 0 | 0 |
| 79 | 631a | 27 | 0 | 0 | 0 |
| 80 | 687b | 27 | 0 | 0 | 0 |
| 81 | 657b | 0 | 0 | 27 | 0 |
| 82 | 645b | 27 | 0 | 0 | 0 |
| 83 | 715a | 0 | 0 | 27 | 0 |
| 84 | 611c | 9 | 33 | 0 | 0 |
| 85 | 685a | 27 | 0 | 0 | 0 |
| 86 | 687c | 27 | 0 | 0 | 0 |
| 87 | 607a | 0 | 50 | 0 | 0 |
| 88 | 727b | 9 | 33 | 0 | 0 |
| 89 | 643i | 0 | 0 | 27 | 0 |
| 90 | 671a | 0 | 0 | 27 | 0 |
| 91 | 611e | 0 | 50 | 0 | 0 |
| 92 | 757b | 0 | 50 | 0 | 0 |
| 93 | 629j | 0 | 33 | 9 | 0 |
| 94 | 769a | 9 | 33 | 0 | 0 |
| 95 | 555a | 0 | 0 | 9 | 100 |
| 96 | 717a | 18 | 0 | 0 | 0 |
| 97 | 645a | 18 | 0 | 0 | 0 |
| 98 | 659a | 9 | 0 | 9 | 0 |
| 99 | 643a | 18 | 0 | 0 | 0 |
| 100 | 611b | 18 | 0 | 0 | 0 |
| 101 | 701a | 0 | 0 | 18 | 0 |
| 102 | 659c | 0 | 0 | 18 | 0 |
| 103 | 595a | 0 | 33 | 0 | 0 |
| 104 | 595c | 0 | 33 | 0 | 0 |
| 105 | 757a | 0 | 33 | 0 | 0 |
| 106 | 641h | 0 | 17 | 9 | 0 |
| 107 | 629h | 0 | 0 | 18 | 0 |
| 108 | 699b | 9 | 17 | 0 | 0 |
| 109 | 643k | 0 | 0 | 18 | 0 |
| 110 | 595d | 0 | 33 | 0 | 0 |
| 111 | 625j | 0 | 0 | 18 | 0 |
| 112 | 695b | 9 | 17 | 0 | 0 |
| 113 | 557a | 9 | 0 | 0 | 0 |
| 114 | 801a | 9 | 0 | 0 | 0 |
| 115 | 641b | 9 | 0 | 0 | 0 |
| 116 | 625a | 9 | 0 | 0 | 0 |
| 117 | 623a | 9 | 0 | 0 | 0 |
| 118 | 661a | 9 | 0 | 0 | 0 |
| 119 | 553a | 0 | 0 | 0 | 100 |
| 120 | 657c | 0 | 0 | 9 | 0 |
| 121 | 629d | 0 | 17 | 0 | 0 |
| 122 | 657e | 0 | 0 | 9 | 0 |
| 123 | 595b | 0 | 17 | 0 | 0 |
| 124 | 613d | 9 | 0 | 0 | 0 |
| 125 | 699a | 0 | 0 | 9 | 0 |
| 126 | 625g | 0 | 17 | 0 | 0 |
| 127 | 701c | 0 | 0 | 9 | 0 |
| 128 | 727a | 0 | 17 | 0 | 0 |
| 129 | 641g | 0 | 0 | 9 | 0 |
| 130 | 639b | 0 | 0 | 9 | 0 |
| 131 | 725a | 0 | 17 | 0 | 0 |
| 132 | 695a | 0 | 17 | 0 | 0 |
| 133 | 643j | 0 | 0 | 9 | 0 |
| 134 | 685c | 9 | 0 | 0 | 0 |
| 135 | 697a | 0 | 0 | 9 | 0 |
| 136 | 699c | 0 | 0 | 9 | 0 |
| 137 | 685d | 0 | 0 | 9 | 0 |
| 138 | 699d | 0 | 0 | 9 | 0 |
| 139 | 785a | 9 | 0 | 0 | 0 |
| 140 | 671c | 0 | 17 | 0 | 0 |
| 141 | 639c | 0 | 0 | 9 | 0 |
| 142 | 625l | 9 | 0 | 0 | 0 |
| 143 | 627j | 0 | 0 | 9 | 0 |
| 144 | 669b | 0 | 0 | 9 | 0 |
| 145 | 643l | 0 | 0 | 9 | 0 |
| 146 | 795a | 0 | 0 | 0 | 100 |
| 147 | 577a | 0 | 0 | 0 | 100 |
